# Functional Characterization of 14 *Pht1* Family Genes in Yeast and Their Expressions in Response to Nutrient Starvation in Soybean

**DOI:** 10.1371/journal.pone.0047726

**Published:** 2012-10-25

**Authors:** Lu Qin, Yongxiang Guo, Liyu Chen, Ruikang Liang, Mian Gu, Guohua Xu, Jing Zhao, Thomas Walk, Hong Liao

**Affiliations:** 1 State Key Laboratory for Conservation and Utilization of Subtropical Agro-Bioresources, Root Biology Center, South China Agricultural University, Guangzhou, China; 2 State Key Laboratory of Crop Genetics and Germplasm Enhancement, College of Resources and Environmental Sciences, Nanjing Agricultural University, Nanjing, China; 3 USDA-ARS, U.S. Pacific Basin Agricultural Research Center, Hilo, Hawaii, United States of America; Centro de Investigación y de Estudios Avanzados del IPN, Mexico

## Abstract

**Background:**

Phosphorus (P) is essential for plant growth and development. Phosphate (Pi) transporter genes in the Pht1 family play important roles in Pi uptake and translocation in plants. Although *Pht1* family genes have been well studied in model plants, little is known about their functions in soybean, an important legume crop worldwide.

**Principal Findings:**

We identified and isolated a complete set of 14 Pi transporter genes (*GmPT1-14*) in the soybean genome and categorized them into two subfamilies based on phylogenetic analysis. Then, an experiment to elucidate Pi transport activity of the GmPTs was carried out using a yeast mutant defective in high-affinity Pi transport. Results showed that 12 of the 14 GmPTs were able to complement Pi uptake of the yeast mutant with *Km* values ranging from 25.7 to 116.3 µM, demonstrating that most of the GmPTs are high-affinity Pi transporters. Further results from qRT-PCR showed that the expressions of the 14 *GmPTs* differed not only in response to P availability in different tissues, but also to other nutrient stresses, including N, K and Fe deficiency, suggesting that besides functioning in Pi uptake and translocation, *GmPTs* might be involved in synergistic regulation of mineral nutrient homeostasis in soybean.

**Conclusions:**

The comprehensive analysis of Pi transporter function in yeast and expression responses to nutrition starvation of *Pht1* family genes in soybean revealed their involvement in other nutrient homeostasis besides P, which could help to better understand the regulation network among ion homeostasis in plants.

## Introduction

As an essential but frequently less available nutrient for plant growth, phosphorus (P) is taken up by plants as orthophosphate (H_2_PO_4_^−^, Pi) mainly through Pi transporters and driven by a proton gradient generated by plasma membrane H^+^-ATPases [1]. In native soil solution, Pi concentration is always less than 10 µM because it is easily bound by either soil organic matter or minerals [2], [3], [4], [5]. Meanwhile, the Pi concentration in the cytoplasm of plant cells is generally greater than 10 mM [6]. Therefore, plants must have specialized transporters to transport Pi from soil solution to plant cells against a large concentration gradient at the root-soil interface. Recent genome sequence analysis and experimental evidence indicated that plants contained a wide variety of Pi transporter families, including Pht1, Pht2, Pht3, Pht4, which were defined by protein sequence, structure, locations and functions [4], [5], [7], [8], [9], [10], [11].

Among the Pi transporter families in plants, Pht1 family is most widely studied. All the members in the Pht1 family have the same predicted structure, including 12 putative membrane-spanning domains, hydrophilic N- and C-terminals, a hydrophilic loop between transmembrane segments (TM) six and seven, a putative glycosylation site in TM10 and two cytoplasmic phosphorylation sites [12]. Since cloning of the first *Pht1* family gene from Arabidopsis [13], many *Pht1* genes have been isolated from a number of plant genomes, including Arabidopsis [14], graminaceous species [15], [16], [17], [18], [19], solanaceous species [20], [21], [22] and legumes [23], [24], [25], [26]. Most Pi transporter genes in the Pht1 family are expressed in roots, while a few are expressed in aerial parts, including leaves, stems, cotyledons, tubers, flowers, grains and seeds [14], [15], [19], [27], [28], implying their potential involvement in Pi internal translocation. Pi transporter genes in the Pht1 family from Arabidopsis and rice, containing 9 and 13 members, respectively, have been comprehensively studied and well characterized [7], [14], [16], [29]. All results indicate that there are distinct functions and different responses to P deficiency among *Pht1* family genes.

In Arabidopsis, eight of nine Pi-transporter genes are expressed in roots. Fusion of the promoter regions from these genes with the GUS reporter gene indicates that four of them are highly expressed in the root epidermis and the expression is enhanced by P deficiency. Additionally, some members are expressed in shoot tissues, such as pollen grains, and thereby implying a wider role in Pi uptake and remobilization [14]. In rice, nine out of thirteen *Pht1* transporter genes are expressed in both Pi-deprived roots and leaves. The transcript levels of *OsPT2, OsPT3, OsPT6* and *OsPT7* are significantly enhanced by P deficiency in roots. The expressions of *OsPT1* and *OsPT8* are abundant in both roots and leaves at two P levels [29], [30]. For legumes, Pi-transporters in the Pht1 family in *Medicago truncatula* have been well studied. Among them, *MtPT1, MtPT2, MtPT3* and *MtPT5* are highly expressed in Pi-deprived roots, but less with addition of high Pi [24]. In *Lotus japonicus*, 3 Pi transporters genes in Pht1 family have been isolated [31].

Soybean (*Glycine max* (L.) Merr.) is one of the most widely grown leguminous crops in the world. However, soybean production is limited by various environmental factors, especially by low P availability in soils [32]. It might help us to find some new approaches to improve the P efficiency of soybean through understanding the detailed characteristics of *Pht1* genes. Compared to the *Pht1* genes in Arabidopsis and rice, much less work has been done in soybean. Recently, two members from the soybean Pht1 family (*GmPT1* and *GmPT2*) were reported to be plasma membrane proteins, and complementation analysis in yeast indicated that they might be constitutively expressed low affinity Pi transporters [23]. Therefore, these two Pi transporters might not be critically involved in response to P deficiency. The discovery and functional characterization of Pi transporters responsible for plant Pi uptake under limited P conditions should be more important to provide a clearer understanding of how plants coordinate the use of Pi to support growth and development.

Plant growth in soils can also be constrained by other nutrients rather than P, and therefore plants might evolve adaptive mechanisms to cope with multiple nutrient stresses. However, only a few studies have reported on interactions among P and other nutrients. For example, coincident potassium (K) and P deprivation induced the transcriptions of a MAP kinase gene, transcription factors and nutrient transporters in tomato [33]. After long term low P treatment, some iron (Fe) and sulfur (S) transporters were up regulated in Arabidopsis [34]. Cross-talks between P and Fe have also been demonstrated in rice [35]. The expression of *AAR6* was up-regulated by N, P, or K deprivation, indicating that shared nutrient signaling transduction pathways might exist in higher plants [36]. As the most responsive gene family to Pi starvation, *Pht1* family genes are likely to be involved in those shared pathways. But up to date, there have been no reports about regulation of *Pht1* family genes by nutrients other than P.

In this study, 14 Pi transporter genes from the *Pht1* family (*GmPTs*) were isolated from the soybean genome. A yeast Pi uptake-defective mutant was used to characterize the Pi uptake kinetics of GmPTs through radioactive ^33^P uptake analysis. Tissue specificity and regulation by P as well as N, K and Fe starvation of all 14 *GmPTs* were analyzed through quantitative RT-PCR (qRT-PCR).

## Results

### Soybean Pi Transporter Genes in the *Pht1* Family

A search of the Phytozome soybean genome database (http://www.phytozome.net/soybean) yielded a total of 14 sequences identified as being related to high-affinity Pi transporters. According to the recommended nomenclature for plant Pi transporters (http://www.botanik.uni-koeln.de/bucher_ppnomenclature.html), these 14 identified genes were named as *GLYma;Pht1;1* through *GLYma;Pht1;14*, with the order being based on their chromosome locations. For simplification, the Pi transporters are called *GmPT1* through *GmPT14* in this report (Table 1). BLAST analysis against the Pfam database (http://www.sanger.ac.uk/resources/databases/pfam.html) showed that they all belonged to the MFS family. The full-length cDNAs and amino acid sequences of these 14 Pi transporters were available in the Phytozome website. All 14 GmPTs exhibited a high degree of homology and were comparable in length, calculated molecular weight and theoretical pI value (Table 1). The distribution of *GmPTs* on soybean chromosomes is uneven and involves only 8 of 20 chromosomes. Chromosome 10 had the highest number of *GmPTs* genes with 4, followed by chromosome 20 with 3 (Table 1).

**Table 1:** Members of Pi transporter genes in the Pht1 family from soybean.

Gene name	Accessionnumber	Locus tag.	aa	kD	pI
*GmPT1*	FJ814697	Glyma02g00840	533	58.47	8.31
*GmPT2*	FJ814696	Glyma03g31950	539	59.27	8.52
*GmPT3*	FJ814701	Glyma07g34870	516	58.31	8.64
*GmPT4*	JQ518269	Glyma10g00950	533	58.42	8.54
*GmPT5*	FJ814694	Glyma10g04230	521	57.31	8.63
*GmPT6*	FJ814693	Glyma10g33020	502	55.44	9.28
*GmPT7*	FJ814695	Glyma10g33030	536	58.73	8.34
*GmPT8*	FJ814700	Glyma13g08720	519	57.64	8.56
*GmPT9*	FJ814698	Glyma14g28780	525	58.24	8.33
*GmPT10*	FJ814699	Glyma14g36650	529	58.19	7.91
*GmPT11*	JQ518270	Glyma19g34710	539	59.27	8.25
*GmPT12*	FJ814692	Glyma20g02660	506	56.78	8.63
*GmPT13*	FJ789662	Glyma20g34610	536	58.63	7.63
*GmPT14*	JQ518271	Glyma20g34620	527	58.11	8.93

Their accession number, locus tag on chromosomes, the respective numbers of amino acids (aa), the calculated molecular mass in kiloDaltons (kD), and theoretical pI value are given.

Like Pht1 transporters in other plant species, the *PT* genes in soybean encode proteins with comparable predicted secondary structures, with each being characterized by 12 hydrophobic domains presumably spanning the plasma membrane. Amino acid sequences of soybean Pi transporter homologs were aligned and compared with each other, and conserved amino acid residues were found (Figure S1). We calculated the relatedness of soybean Pht1 transporters using the DNAMAN computer program, with the results suggesting that the percentage of identical protein sequences ranges from 48% to 99% (Table S1). The highest percentage of amino acid sequence identity was found between GmPT2 and GmPT11 (99.27%), followed by GmPT6 and GmPT14 (98.23%). The lowest identity was observed between GmPT3 and GmPT10 (47.63%). GmPT3 and GmPT12 had a very high amino acid sequence identity of 93.89%, but both proteins had comparatively low amino acid sequence identity with all the other 12 Pht1 Pi transporter proteins.

### Phylogenetic Analysis of GmPTs

**Figure 1:**
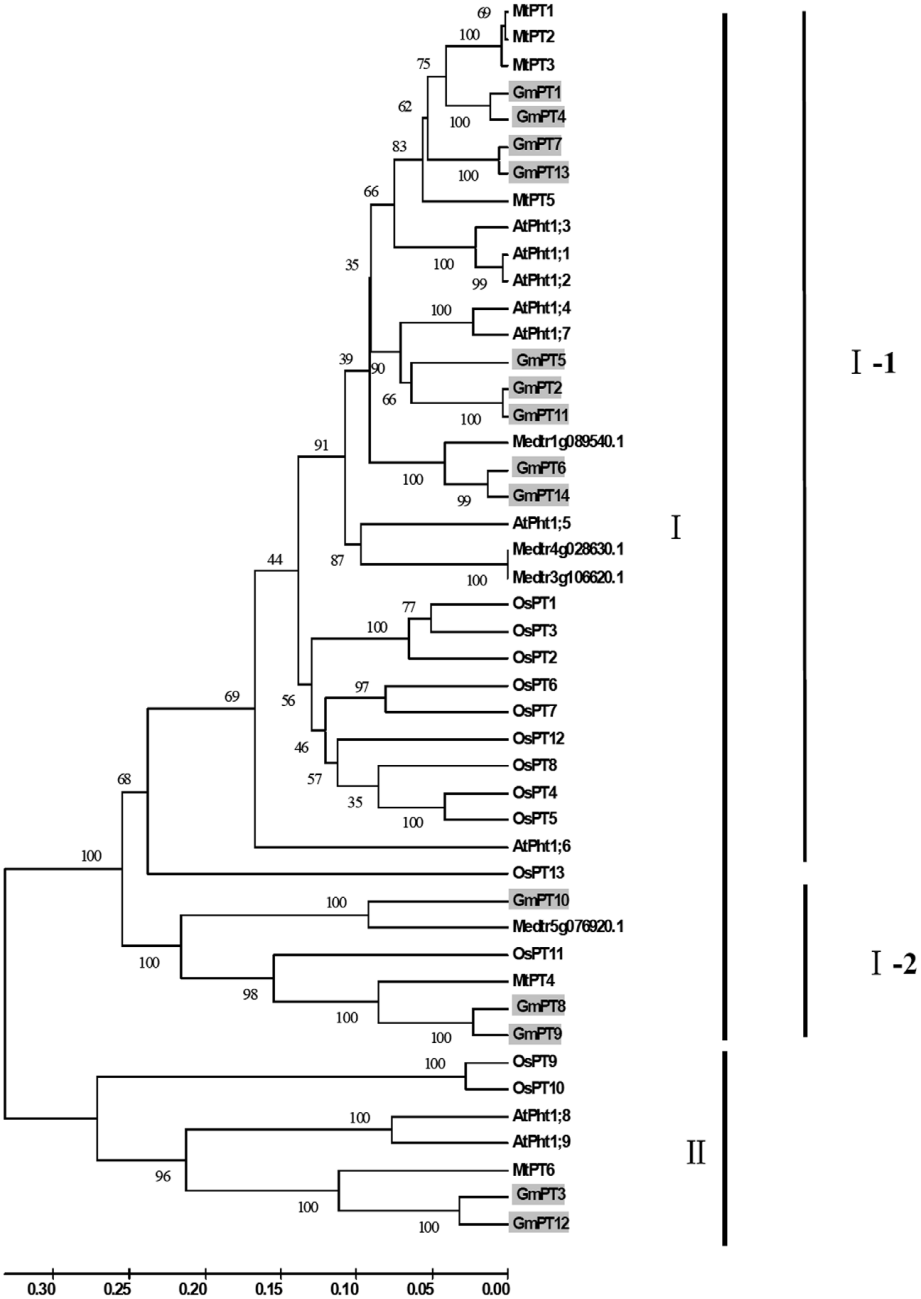
Phylogenetic tree of soybean, Arabidopsis, rice and Medicago plant Pi transporter proteins in Pht1 family. Transporters and corresponding plant species are as follows: rice (*Oryza sativa*), OsPT1 through OsPT13 [16]; Arabidopsis (*Arabidopsis thaliana*), AtPht1;1 through AtPht1;9 [14]; Medicago (*Medicago truncatula*) MtPT1 through MtPT6 [68] and other four PT proteins obtained in Phytozome (http://www.phytozome.net/medicago), soybean (*Glycine Max*), GmPT1 through GmPT14 (this work).

Combining GmPT1 through GmPT14 with Pi transporter protein sequences in rice, Arabidopsis and *Medicago truncatula,* we constructed a phylogenetic tree using neighbor-joining analysis in the MEGA 4.1 program (Figure 1). The phylogenetic results demonstrated that like the other three species, soybean GmPTs were divided into two distinct groups with strong bootstrap support. Group I contained 12 of the 14 GmPTs, 9 of 10 MtPTs, 7 of the 9 AtPhts and 11 of the 13 OsPTs. Group I could be further divided into two subgroups: I-1 and I-2. Subgroup I-1 contained 9 GmPTs, 7 MtPTs, 7 AtPhts and 10 rice Pi transporters. It is interesting to find that in subgroup I-2, GmPT8, GmPT9 and GmPT10 were clustered with the mycorrhiza-specific OsPT11 from rice [16] and MtPT4 from *Medicago truncatula* [24], [25], and no Arabidopsis AtPTs, implying possible roles for these three GmPTs in Arbuscular Mycorrhizal (AM) symbiosis. Group II only included a few Pi transporters, a pair from soybean (GmPT3 and GmPT12), a pair from Arabidopsis (AtPht1;8 and AtPht1;9), MtPT6 from *Medicago truncatula* and a pair from rice (OsPT9 and OsPT10). Both group I and group II contained Pht1 family Pi-transporters from all four species, indicating that divergence of Pi-transporters preceded divergence of dicots and monocots. By exploring the intron (the noncoding sequence between two coding sequences within a gene) and exon (the protein-coding region in the DNA) structures of GmPTs, we found the genes from the same group had the similar intron/exon structures, such as GmPT3 and GmPT12 in group II, their structures were totally different from those in Group I by characterized with three exons and two long introns, indicating the divergence of structures and functions of different GmPTs (Figure S2).

### Analysis of Pi Transport Activities of GmPTs in a Yeast Strain Defective in Pi-uptake

To analyze and compare the Pi transport activities of all the GmPTs, their coding regions were separately cloned into a yeast expression vector (p112A1NE), under the control of the yeast alcohol dehydrogenase promoter. The constructs were separately introduced into a yeast Pi transport mutant MB192, which lacks the function of the high-affinity Pi transporter gene *PHO84*. An empty vector was also transformed to be used as a control.

Because the Pht1 transporters are members of the H^+^/Pi symporter family, the pH dependence of GmPTs during Pi transport was assayed by measuring the optical density of the yeast cell lines at pH values ranging from 4 to 8. The pH optima for most of the yeast mutant cells carrying p112-GmPTs was 6, in comparison to the wild type, in which the pH optima ranged from 4 to 6. Therefore, the pH value was set as 6 in the subsequent studies.

**Figure 2:**
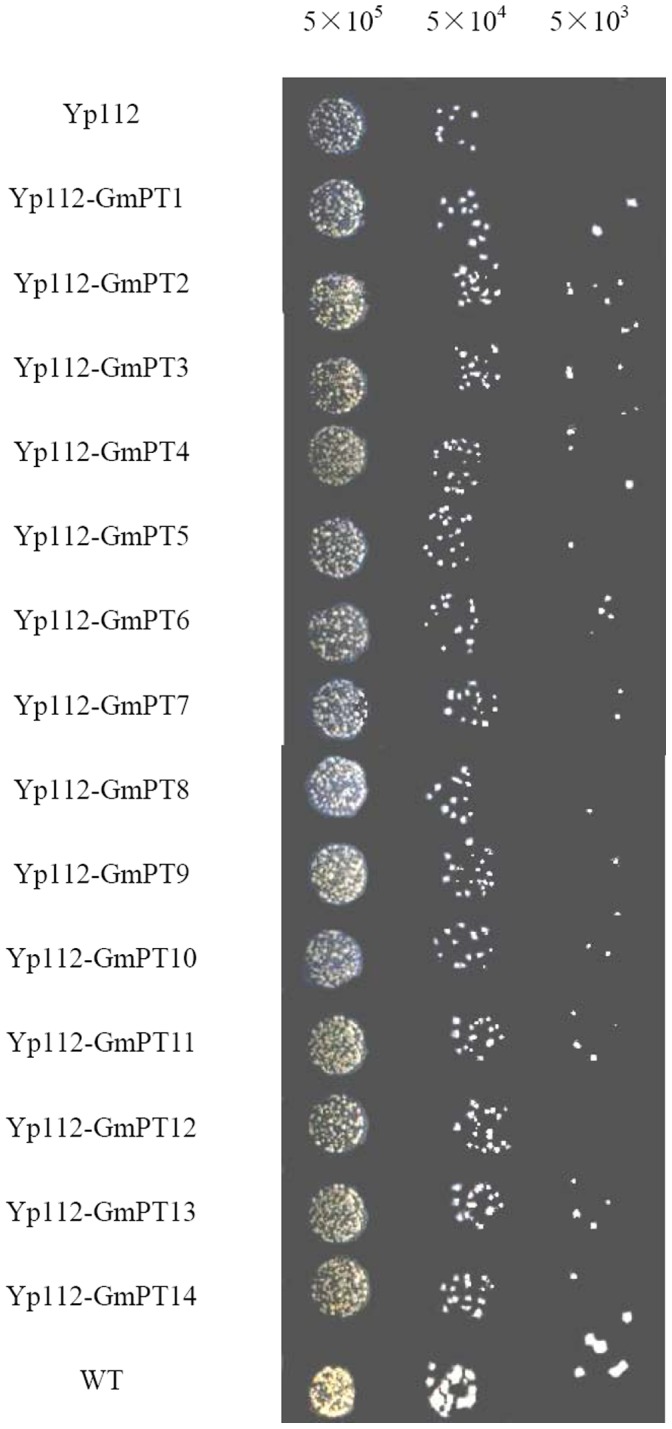
Complementation of a yeast inorganic phosphate (Pi) transport mutant by *GmPTs* genes. Yeast MB192 cells harboring either an empty expression vector (control) or the candidate Pht1 ORF(open reading frame), transformants were grown in YNB medium to an OD600 ≈0.8, then washed by 3% glucose with centrifugation at 1500 g, 4°C, and suspended in phosphate free YNB medium to OD600 ≈1.0. Different number cells (5×10^5^, 5×10^4^, 5×10^3^) were applied to Pi-limiting medium (20 µM, pH 6.0) then incubated at 30°C for 3 d.

The yeast MB192 strain grows poorly when supplied with limiting amounts of Pi. As shown in Figure 2, under low P conditions (20 µM), all the *GmPT* transformants grew much better than the control, suggesting that GmPTs can complement the yeast Pi-uptake mutant under Pi-limiting conditions (Figure 2).

To make sure the effects of the expressions of *GmPT* genes in yeast on the growth of transformed yeast cells are Pi-deficiency dependent, but not just because of the expressions of *GmPT* genes, we analyzed the kinetic growth of Yp112-GmPTs transformants, wild type and empty vector control in normal YNB liquid medium with 2 mM Pi, and found that there were no significant growth differences between empty vector control and all the 14 Yp112-GmPTs transformants (Figure S3D), But under certain low P conditions, Yp112-GmPTs transformants always grew better than the empty vector control (Figure S3A, B, C), indicating the complementation of GmPTs was really Pi-deficiency dependent.

**Figure 3:**
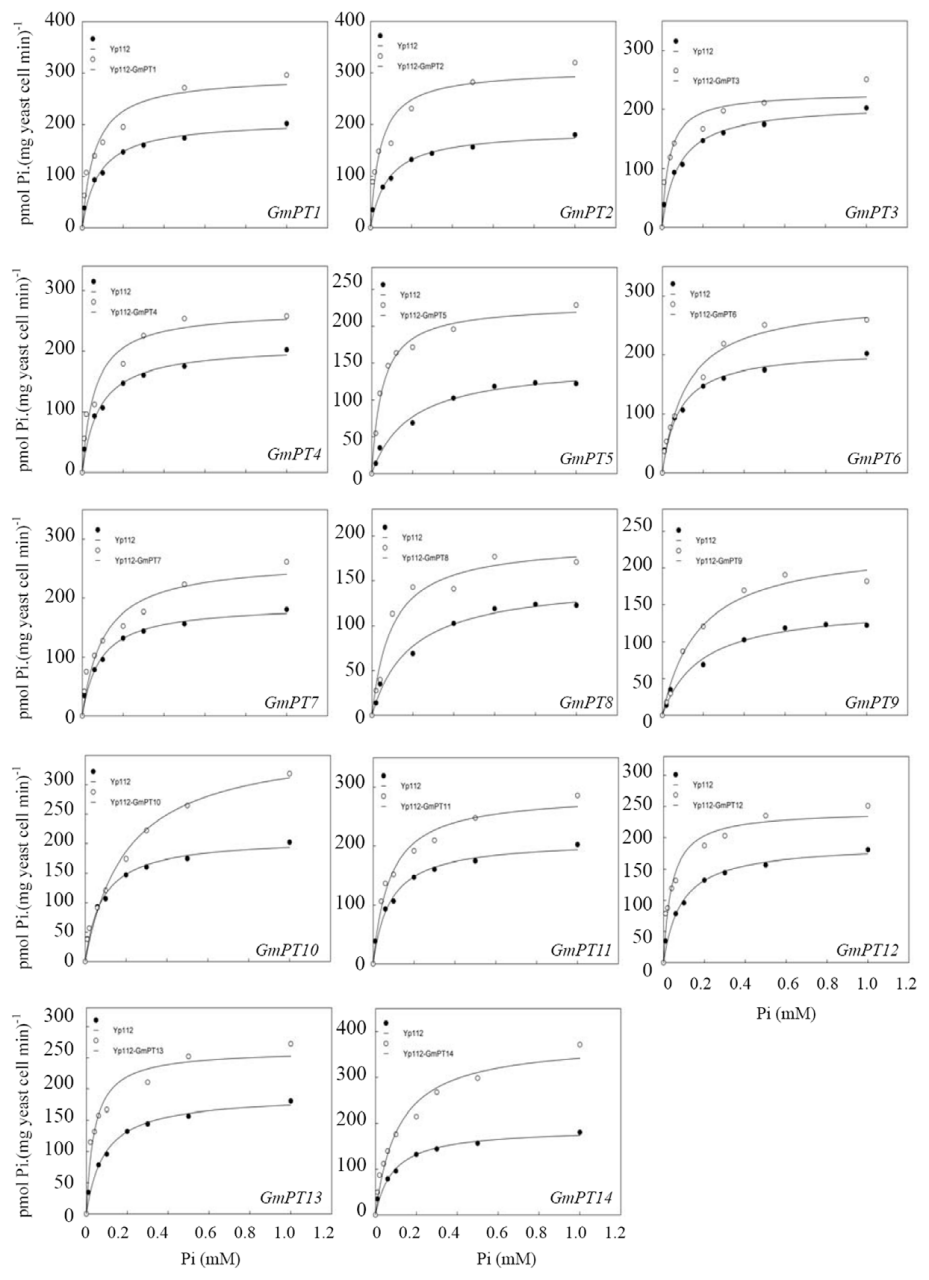
Kinetic analysis of inorganic phosphate (Pi) uptake in yeast. The non-linear regression of total Pi uptake by strain Yp112-GmPTs versus external Pi concentration at pH 6 were used to estimate the apparent *Km* value for Pi uptake. All the results were calculated from the three independent experiments.

Furthermore, in order to determine the kinetic properties of GmPTs,^ 33^P was employed in Pi-uptake experiments using transformed yeast cells. A Line weaver-Burk plot indicated that Pi uptake mediated by 12 *GmPT* genes followed Michaelis-Menten kinetics (Figure 3) with the estimated *Km* values ranged from 25.70±1.63 µM to 116.30±10.00 µM (mean±SE) (Table 2), while only GmPT6 and GmPT10 showed no difference when Pi concentration in the growth medium was less than 0.1 mM. The results from kinetic studies above demonstrated that most of the GmPTs were high-affinity Pi transporters.

**Table 2:** Kinetic parameter estimates of GmPTs-mediated inorganic phosphate (Pi) transport.

Yeast	*Km*(µM)	*Vmax*(µM/g [dry weight]·min)
Yp112-GmPT1	67.30±15.60	298.30±24.40
Yp112-GmPT2	44.00±12.90	295.00±20.90
Yp112-GmPT3	30.00±8.60	226.70±16.30
Yp112-GmPT4	65.30±13.30	282.30±43.28
Yp112-GmPT5	25.70±1.63	231.30±18.80
Yp112-GmPT6	153.00±44.00	281.70±37.60
Yp112-GmPT7	105.30±20.00	247.00±24.50
Yp112-GmPT8	46.00±5.60	231.50±5.50
Yp112-GmPT9	88.00±8.90	240.30±7.10
Yp112-GmPT10	231.00±15.60	400.00±25.00
Yp112-GmPT11	79.00±8.30	271.30±45.00
Yp112-GmPT12	45.30±14.53	245.30±15.60
Yp112-GmPT13	32.30±1.60	265.70±32.50
Yp112-GmPT14	116.30±10.00	373.00±14.10
Yp112	127.00±8.50	154.70±3.60

*Km* and *Vmax* for yeast strain MB192 expressing the indicated *GmPTs* or carrying the empty expression vector (control) were determined at pH 6.0. The GmPTs mediated ^33^Pi uptake velocities, calculated according to their total Pi transport, following the Michaelis-Menten kinetics equation. Values shown are means ± SE for three independent experiments.

### Expression Patterns of *GmPTs* as Regulated by P Availability in Different Tissues

**Figure 4:**
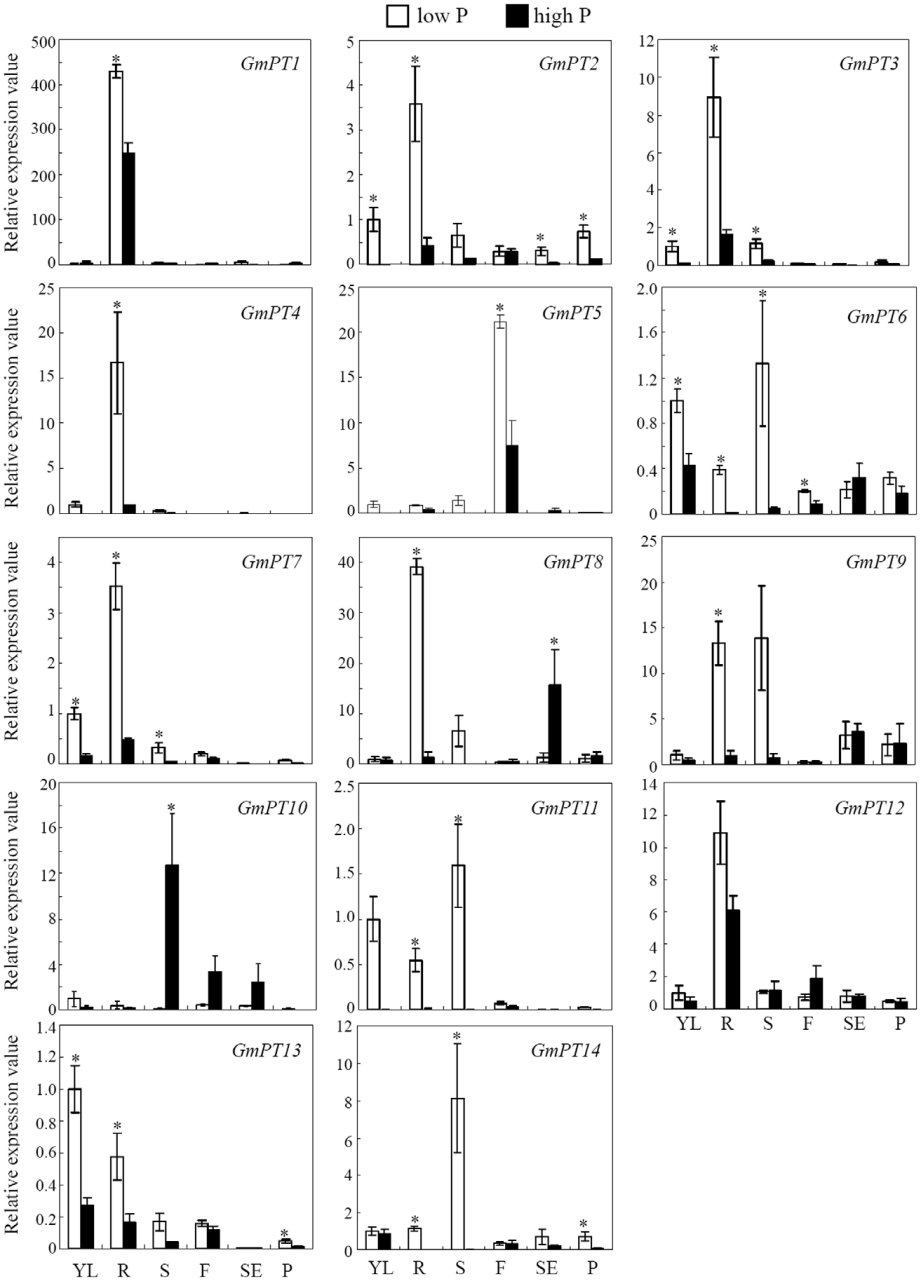
Spatial Expression pattern analysis for the 14 *GmPTs* as related to P availability. Plants were grown on low P (added 5 µM P as KH_2_PO_4,_ open bars) and high P (added 500 µM P as KH_2_PO_4,_ closed bars) conditions. Young leaves (YL), roots (R), stems (S) and flowers (F) were sampled 18 days after treatment initiation, and young pods (P) and seeds (SE) were sampled 29 days after treatment initiation. Each bar is the mean of three biological replications with standard error. Note: different scales are used in the graphs; asterisks indicate significant differences of *GmPTs* expression in certain tissues under low P and high P conditions in *t*-tests.

According to the nucleic acid sequences (Text S2), we designed gene-specific primers for the 14 *GmPTs* genes (Table S2). Tissue specificity and P response of the *GmPTs* were investigated using qRT-PCR. The main expression pattern under high P conditions was consistent with the results from the two soybean transcriptome atlases (Table S3) [37], [38], such as the expression levels of most *GmPTs* were low, and roots had the highest number of *GmPTs* genes to express in, indicating the important roles of Pi transporters in Pi transport and absorption in roots; *GmPT5* mainly expressed in flowers, implying possible functions in Pi transport for source to sink (Figure 4).

**Figure 5:**
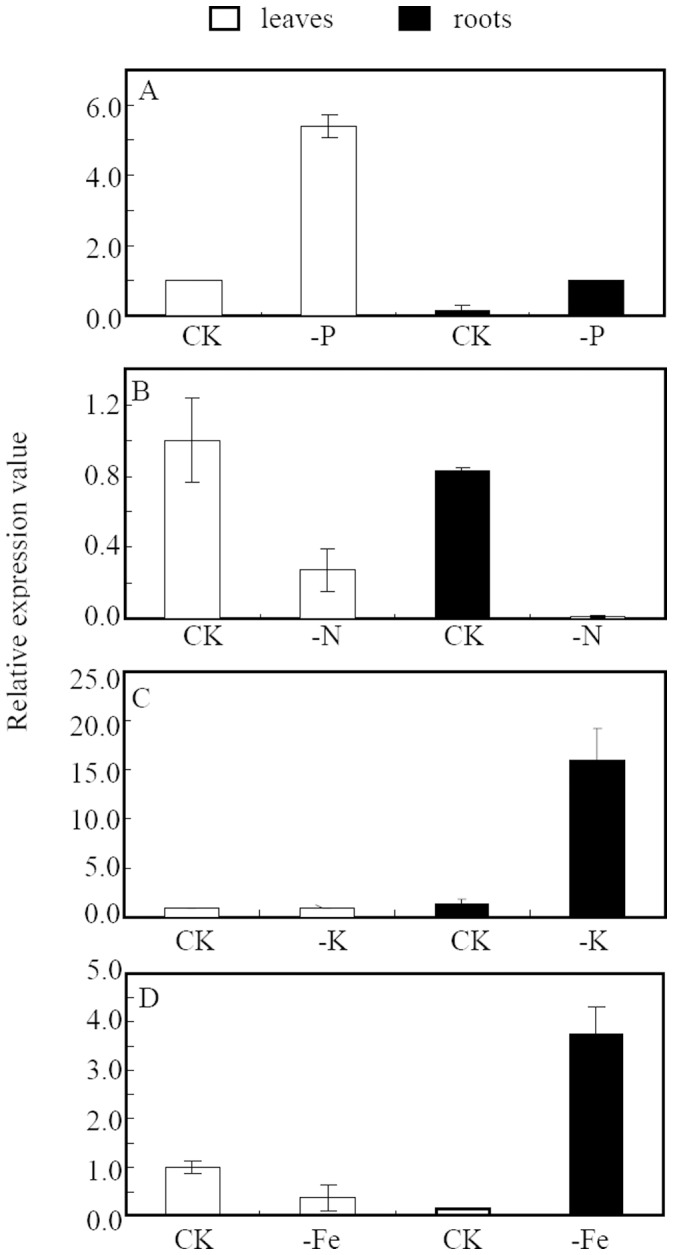
Expression of P, N, K or Fe responsive genes to different nutrient stresses. 14-day old soybean seedlings were treated with N (−N), K (−K) and Fe (−Fe) deficiencies (see Experimental procedures for details). Seedlings grown under normal solution were used as controls (CK, added 500 µM P as KH_2_PO_4_). The expression levels in shoots and roots were analyzed by quantitative real-time PCR. Soybean gene *PLDZ* (Glyma20g38200) was used as low P responsive gene (A), *NiR* (Glyma02g14910) for low N treatment (B), *HAK* (Glyma3g42480) for low potassium treatment (C) and *IRT* (Glyma07g34930) for low Fe treatment (D), respectively. Each bar was the mean of three biological replications with standard error.

The expression of *GmPLDZ* (Glyma20g38200) which has been well demonstrated as a Pi starvation induced gene [39], was also analyzed presently. The results found that the *GmPLDZ* expression was highly enhanced under low P conditions, especially in leaves (Figure 5A), proving that the P treatments in the present study were sufficient for analyzing the responses of Pi starvation induced genes. Like Pi transporters in other plant species, the expressions of *GmPTs*, except *GmPT10*, were highly induced by P deficiency (Figure 4). Seven, including *GmPT1*, *GmPT2*, *GmPT3*, *GmPT4, GmPT7 GmPT8* and *GmPT12*, were predominantly expressed in roots. *GmPT5* and *GmPT14* were mainly expressed in flowers and stems, respectively. *GmPT9* and *GmPT13* were highly induced in roots and stems, and young leaves and roots, respectively. *GmPT6* and *GmPT11* were expressed highly in young leaves, roots and stems (Figure 4). In addition, Pi starvation induced *de novo* synthesis of *GmPT3* and *GmPT4* in roots and *GmPT11* in young leaves, roots and stems. In contrast, *GmPT10* was expressed in stems, flowers and seeds at high P level. Finally, low or even undetectable expressions of *GmPTs* were observed in pods (Figure 4).

### Responses of *GmPTs* to Nitrogen, Potassium and Iron Deficiency

To examine the potential induction of *GmPT* genes by other nutrient deficiencies, transcript abundance was assessed by qRT-PCR in soybean plants separately grown in nutrient solution deficient in nitrogen (N), potassium (K) or iron (Fe) for 14 days. Three marker genes, including *GmHAK* (Glyma3g42480, a K transporter) and *GmIRT* (Glyma07g34930, an iron transporter) which had been known respectively enhanced by K or Fe deficiency [40], [41], and *GmNiR* (Glyma02g14910, a nitrite reductase gene) which was repressed by N deficiency [42], were used to monitor the treatmental conditions. As expected, the expression of *GmHAK*, *GmIRT* and *GmNiR* was significantly regulated by K, Fe or N deficiency, respectively (Figure 5B, C, D).

**Figure 6:**
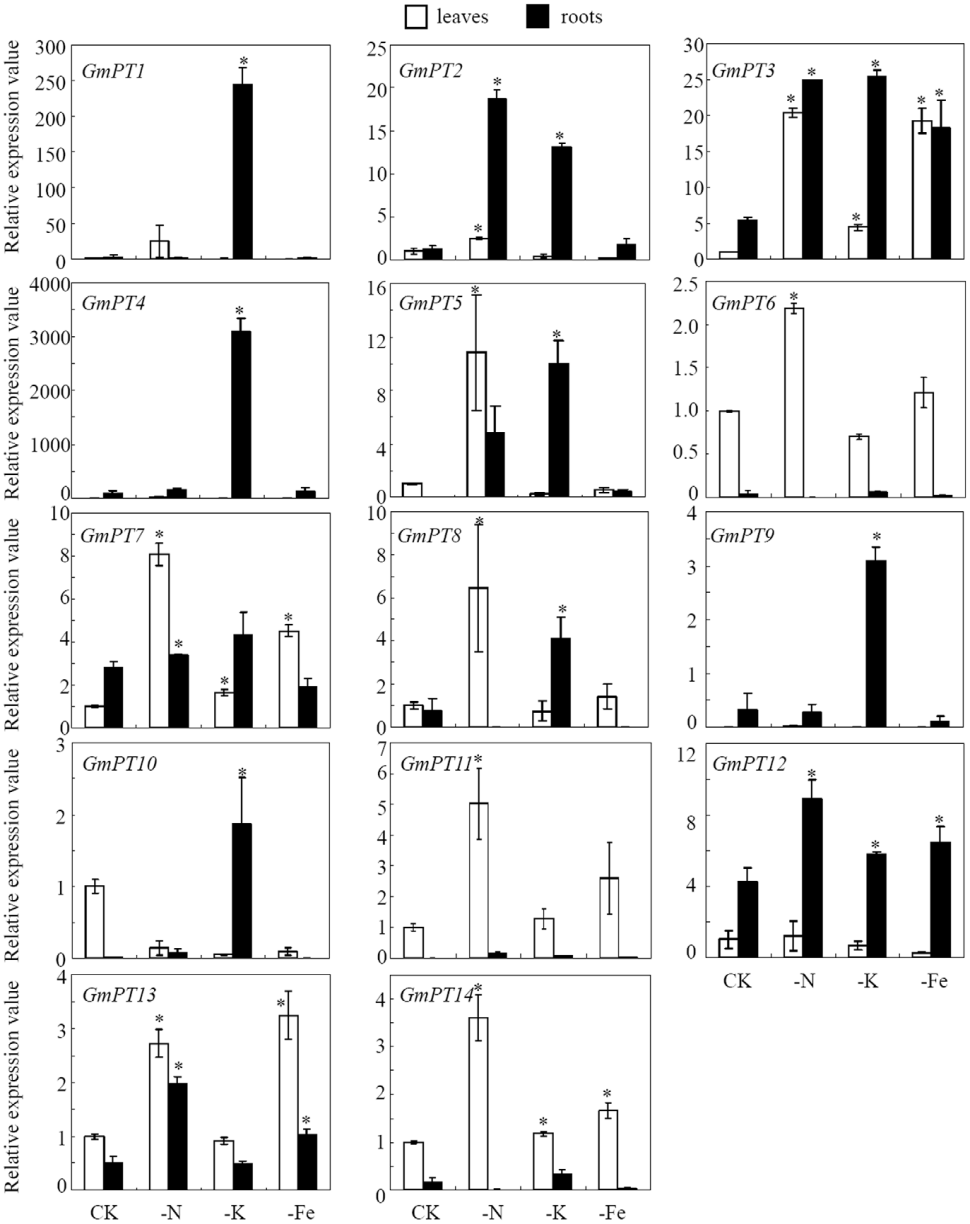
Responses of *GmPTs* to different nutrient stresses. Ten-day old soybean seedlings were treated with N (−N), K (−K) and Fe (−Fe) deficiencies (see Experimental procedures for details). Seedlings grown under normal solution were used as controls (CK, added 500 µM P as KH_2_PO_4_). Asterisks indicated the significant differences of *GmPTs* expression between nutrients deficient stresses and normal conditions in Student’s t-tests.

The expressions of *GmPT* genes in response to N, K, or Fe deficiency were shown in Figure 6. The 14 Pi transporter genes had very low expressions in leaves and roots under normal conditions. All of the gene expression levels were modified by one or more of the three stresses in different tissues. Under N deficiency conditions, the expressions of 10 genes were up-regulated more than 2-fold. Among them, *GmPT6, GmPT7, GmPT8, GmPT11* and *GmPT14* were increased in leaves, and *GmPT12* was in roots. N deficiency enhanced the expressions of *GmPT2, GmPT3, GmPT5* and *GmPT13* in both leaves and roots. K deficiency enhanced the expressions of 8 *GmPTs* genes. *GmPT3* was up-regulated in both leaves and roots, while the expressions of the other 7 *GmPTs*, including *GmPT1, GmPT2, GmPT4, GmPT5, GmPT8, GmPT9* and *GmPT10,* were only enhanced in roots. Under Fe deficiency, *GmPT3, GmPT12* and *GmPT13* were up-regulated. The expression levels of the remaining *GmPT* genes were not altered significantly under Fe deficiency. Interestingly, *GmPT3* was the only one gene with expression up-regulated by N, P or K deficiency simultaneously in both leaves and roots (Figure 6), suggesting that *GmPT3* might be involved in an universal regulation network in response to multiple nutrient deficiencies.

## Discussion

Phosphorus is particularly critical for legume growth due to the huge demands for protein and oil synthesis, as well as, for biological N_2_ fixation [43]. However, low P availability is a worldwide constraint for crop growth in most soils. Therefore, to understanding Pi uptake and translocation in soybean can dramatically facilitate soybean adaptation to the low P soils. Phosphate transporter genes in the *Pht1* family have been reported playing important roles in Pi uptake and translocation in plants [14], [16], [17], [19], [22], [24], [44], [45]. To date, information on this family is limited in soybean. In the current study, we identified and isolated 14 members of PTs in the Pht1 family from the soybean genome, and characterized their functions in Pi uptake using yeast mutants defective in high affinity Pi transport. The expression patterns in terms of tissue specificity, responses to P deficiency and other nutrient stresses were also analyzed.

### Soybean Pi Transporter Genes in *Pht1* Family

Like *Pht1* family genes in other species, soybean PTs in the Pht1 family exhibited a high degree of homology, characterized by high protein identity (Table S1) and similar hydrophobic domains presumably spanning the plasma membrane (Figure S1). Interestingly, phylogenetic analysis of GmPT1 through GmPT14, along with Pi transporter protein sequences from *Medicago truncatula*, Arabidopsis, and rice, showed a similar pattern of evolutionary divergence within dicotyledons (Arabidopsis, Medicago and soybean) and monocotyledons (rice). These phylogenetic relationships among the GmPTs (Figure 1), may reflect biochemical and functional differences among different types of PTs.

With well-supported bootstrap values, GmPT8 and GmPT9 were clustered with the mycorrhiza-specific OsPT11 from rice [16] and MtPT4 from *Medicago truncatula*. Since OsPT11 is specifically induced by AM symbiosis, and MtPT4 is critical for AM symbiotic Pi transport and development, the absence of MtPT4 will lead to the inability of the fungus to proliferate within roots, and termination of symbiosis [25]. We speculate that, given the phylogenetic proximity, GmPT8 and GmPT9 are likely to play important roles in the symbiosis between soybean and AM fungus. GmPT10 was classified into the same subgroup with GmPT8 and GmPT9, though with a weaker bootstrap value than that between GmPT8 and GmPT9, implying a possible role for GmPT10 in symbiosis with AM fungi, but its functions might be different from those of GmPT8 and GmPT9 in AM roots.

GmPT3 and GmPT12, which had relatively lower protein sequence identity with the other GmPTs (Table S1), were classified into the group II (Figure 1) of *Pht1* genes. By exploring gene structures, we found these two genes shared a similar and slightly unusual structure with a relatively large intron in the coding sequence and more exons than the other GmPTs, which had only one or two exons (Figure S2). These structural differences might be related to the special functions of GmPT3 and GmPT12 differing from other GmPTs. Recent reports have indicated that the size of exon and intron, and their intergenic distance are correlated with gene expression levels and expression breadth in soybean [46]. The relationship between gene structures and functions of GmPTs needs to be further analyzed.

### Functions of GmPTs in Pi Uptake

Three systems have been well accepted to functionally characterize plant Pi transporters, including the complementation of yeast mutants defective in Pi transport, monitoring Pi uptake using *Xenopus laevis* oocytes and ectopic expression of Pi transporters in plant suspension cells [15], [28], [30], [47], [48], [49]. The yeast mutants defective in Pi transporters are most widely used for characterizing the kinetic properties of many PTs from different plants. With complemented yeast mutants, kinetic studies allow for quantifying the affinity of transporters [15], [47], [50]. However, few whole Pht1 families in crops have been functionally analyzed [15], [30]. The yeast Pi transport mutant MB192, which lacks function of the high-affinity Pi transporter gene *PHO84*, has been used to analyze Pi transporters from several crop species [30], [51]. In the present study, we used the MB192 yeast system to work on kinetic analysis of all 14 GmPTs, and found that 12 of 14 GmPTs acted as high-affinity Pi transporters (Table 2, Figure 3), implying their functions in high-affinity uptake and transport of Pi from low P soils. However, among the five reported *Pht1* family genes in *Medicago truncatula*, only MtPT5 showed high affinity for Pi uptake [24], [26]. The possible reason why we could identify so many high-affinity Pi transporters here using the yeast system is that due to the existence of native Pi transport systems, the expression of high-affinity Pi transporters in yeast cells might display altered transport properties. Some high-affinity Pi transporters, such as AtPHT1;1 and HvPHT1;1, were not able to complement the yeast mutant. We also found that among the tested GmPTs, although GmPT6 and GmPT10 could complement the Pi absorption of the yeast mutant MB192 to a certain extent (Figure 2), but their Km values were higher than that of the tested mutant based on the kinetic analysis (Figure 3, Table 2). Therefore, we still characterized them as low-affinity Pi transporters. However, there is not a defined Km value for categorizing the Pi affinity of PTs, and the external Pi concentrations used here were limited, further works are needed to test weather these two transporters have dual-affinity for Pi uptake.

### Characterization of the *GmPTs* in Response to P Availability

Recently, two soybean transcriptome atlases providing a record of high-resolution soybean gene expressions have been reported by two groups using next generation sequencing technique [37], [38]. The expression patterns of the 14 *GmPTs* with normal Pi supply in the present study are well consistent with those atlases. Most of the *GmPTs* showed low expressions in most tissues, and some were undetectable (Figure 4) [37], [38]. Differently, we also found that *GmPT10* was mainly expressed in stems, flowers and seeds in high P conditions, implying it might be a low-affinity Pi transporter functioning in internal Pi translocation. The differences among our results and the previously reported transcriptome atlases might be due to the different sampling stages and growth systems. In addition to work on normal growth conditions, we tested the responses of the 14 *GmPTs* to Pi starvation, and found that most of the *Pht1* genes had elevated transcript levels under low P conditions, while a few of them were differentially expressed between roots and aerial parts of the plants as previously reported [13], [14], [17], [24], [28], [47], [50]. Thirteen *GmPTs* were induced or increased under low P conditions in soybean (Figure 4), indicating their roles in soybean adaptation to low P availability. Six *GmPTs* were mainly expressed in roots, and the others were differently expressed in stems, flowers and seeds. As a whole, these results suggest pivotal functions for *GmPTs* in both Pi acquisition and translocation. Low or no expressions of *GmPTs* were detected in pods (Figure 4), indicating rare Pi exchange occurred or other Pi transporters function in this organ. The differential expressions of individual genes in different plant organs imply that there might be additional functions for the respective genes other than Pi uptake.

### Involvement of *GmPTs* in N, K and Fe Signals

Previous reports strongly suggest that there might be crosstalks among ion signals in response to different nutrient stresses, the expressions of ion transporters might be involved in a process that facilitates mineral nutrient homeostasis [52], [53], [54], [55]. Wang *et al* [55] used a high-density array from tomato roots to test the expression profiling of 1,280 genes in N, K or Fe deficiency, and found the expressions of some Pi, K and Fe transporter genes were up-regulated by all three nutrient deficiencies. This suggests some transporter genes might be involved in the coordinated and coregulated uptake of these essential nutrient elements. Nitrogen, P, K, and Fe deficiencies are well known as important limiting factors to agricultural production [56], but no research has been reported on the responses of Pi transporters to N, K or Fe deficiency. Therefore, studying expressions of *GmPTs* genes under nutrient deficiencies other than P deficiency could reveal possible functions of Pi transporters in multiple mineral nutrient homeostasis.

The rice *PHO1* gene family has been well documented to play important roles in Pi translocation from roots to shoots. Furthermore, one of the *PHO1* family genes was up-regulated by N starvation [57], implying crosstalks exsiting between P and N signals in rice. Recent work on the *NLA* (N limitation adaptation) gene in Arabidopsis indicates that there is an antagonistic crosstalk between N and P deficiency [58]. It has been reported that *NLA* is involved in adaptive responses to low N conditions, where *nla* mutant plants display abrupt early senescence. Further analysis found the two suppressors of the *nla* mutation can impart the *nla* mutant phenotype to *NLA* wild type plants, and these suppressors have been proved to be the Pi transport-related genes, PHF1 and PHT1.1. In addition, *NLA* expression is regulated by the low-Pi induced microRNA miR827, suggesting that Pi transporters could directly affect plant N nutrition. In the present study, we found that most of the soybean *PTs* genes were significantly up-regulated under N deficiency, indicating that at least some of the Pi transporters are involved in N signaling pathways in soybean. Increased expression of a high-affinity Pi transporter gene was also found under low K in barley [59], showing the existence of interactions between P and K signals. This is also supported by our results in which 8 *GmPTs* were regulated by K deficiency (Figure 6). The underlying molecular mechanisms and pathways involved in these crosstalks need to be further researched.

Iron is the most studied nutrient element for interactions with P due to their strong precipitation [60]. It is well accepted that P and Fe deficiency have similar effects on the differentiation of epidermal cells, and subsequently affect root growth, including lateral root and root hair formation [61], [62], [63]. In Arabidopsis, primary root growth was inhibited by P deficiency due to Fe toxicity in the root tip [64]. But no P and Fe interactions related to Pi transporters has been reported. Here we found that the expressions of 5 *GmPTs* highly responded to Fe deficiency (Figure 6), and thus provided the first evidence that Pi transporter genes might be involved in interactions between P and Fe signaling.

All together, we conclude that there are strong interactions among N, P, K and Fe signals, and Pi transporter genes might be involved in cross-talks for sensing the changes of N, P, K and Fe status in soybean.

## Materials and Methods

### Computational Identification of the Pht1 Family Members of Pi Transporters in Soybean

The nucleic acid sequences of all 9 and 13 members of PTs in the Pht1 family in Arabidopsis [14] and rice (*Oryza sativa*) [16], respectively, were used as query sequences to BLAST search the Phytozome soybean genome database (http://www.phytozome.net/soybean). The predicted sequences revealed there were in total 14 members of the *Pht1* gene family in soybean. The nucleic acid and amino sequences of the 14 Pi transporters were retrieved from the Phytozome website (Text S1, S2). Protein molecular weights and theoretical pI values were calculated using Compute pI/Mw tool (http://www.expasy.org/tools/pi_tool.html). Sequence identity was performed using DNAMAN version 6.0 (Lynnon Biosoft Company). Sequence alignment was performed with ClustalW [65]. The membrane-spanning domains of GmPT1 through GmPT14 were predicted by TopPred (http://www.cbib.u-bordeaux2.fr/pise/toppred.html). A phylogenetic tree based on entire protein sequence alignments using ClustalW was constructed by the neighbor-joining method with 1000 bootstrap replicates in the MEGA 4.1 program (http://www.megasoftware.net/mega4/mega41.html). Complete deletion was used to deal with gaps or missing data in sequences. The distance between sequences was estimated after Poisson correction. The distance between sequences was estimated after Poisson correction. Primers in Table S4 and Table S5 were used to amply the ORF and DNA sequences of *GmPTs*, respectively, the gained ORF and DNA sequences were used to construct gene structures through Gene Display Server (http://gsds.cbi.pku.edu.cn/index.php).

### Yeast Manipulations

*Saccharomyces cerevisiae* MB192 (*MATa* pho3–1 pho84::HIS3 ade2 leu2-3, 112 his3-532, trp1-289 ura3-1, 2 can1) defective in the high-affinity Pi transporter gene *PHO84* by insertion of an *HIS3* DNA fragment [66], and the expression vector, p112A1NE, were used to functional complementation assay of *GmPTs* following the protocol described previously. The full coding regions of each of the 14 *GmPTs* were produced by RT-PCR. PCR primers were designed to introduce unique restriction sites at the 5′ and 3′ ends of the genes (Table S4). The cDNA amplicons were cloned into the yeast expression plasmid p112A1NE to create Yp112-GmPTs. According to Dohmen *et al* [67], these constructs and empty vector (as control) were transformed into the MB192. Yp112-GmPTs transformants were tested for their ability to complement the growth defect of MB192. Transformed yeast strains grew in yeast nitrogen base liquid (YNB) medium to the logarithmic phase (when the absorbance at 600 nm was 0.8 at 30°C), and then were harvested and washed (centrifugation at 1500 g, 4°C, 5 min) three times with Pi-free YNB medium (containing an equivalent concentration of potassium chloride rather than potassium phosphate). The collected pellets were suspended in the same medium and incubated at 30°C until absorbance at 600 nm was 1.0, as preparation for the next experiments. For pH-dependent Pi uptake experiments, different extracellular pH values in the range of 4.0–7.0 were used. For ^33^P uptake experiments in yeast, about 1 mg fresh yeast cell samples were used following the modified method as previously described [15]. Eight different concentrations of Pi (10 µM, 20 µM, 60 µM, 100 µM, 200 µM, 300 µM, 500 µM and 1000 µM) were used to assay Pi uptake by intact *Saccharomyces cerevisiae* cells by the addition of 2 µL of [^33^P]orthophosphate to 50 µL aliquots of cells, were incubated with shaking at 30°C for 5 min, Pi uptake was stopped by the addition of 1 mL cooled tris-succinate, harvested, then suspended in 25 mM Tris-succinate (pH 6) solution, and washed three times (centrifugation at 1500 g, 4°C, 5 min) with 3% glucose. Radioactivity was measured by a Beckman LS 6500 Scintillation Counter. The data were analyzed using the software SIGMAPLOT (v10.0) to determine the *Km*. For growth experiments on YNB solid medium, different numbers of transformant cells (5×10^5^, 5×10^4^, 5×10^3^) calculated by blood cell counting plate methods were plated on pH 6.0 Pi limited (20 µM) YNB agar plates and culture at 30°C, 3 days. There were three independent biological experiments for each measurement. For growth experiments in YNB liquid medium, 100 µL cells in the logarithmic phase (OD600≈0.90) were subjected to 3.5 mL YNB liquid medium with different Pi concentrations and incubated at 30°C, OD 600 were measured every 5 hours up to 25 hours.

### Plant Materials and Growth Conditions

Soybean cv. HN66 was employed in this study. For expression analysis of *GmPTs* in different tissues responding to P availability, soybean plants were nutrient solution cultured in a greenhouse. One week after germination, the seedlings were transplanted into the full-strength nutrient solution containing 500 µM KH_2_PO_4_, 3000 µM KNO_3_, 2000 µM Ca(NO_3_)_2_, 250 µM MgSO_4_, 25 µM MgCl_2_, 12.5 µM H_3_BO_3_, 1 µM MnSO_4_, 1 µM ZnSO_4_, 0.25 µM CuSO_4_, 0.25 µM (NH_4_)_6_Mo_7_O_24_ and 25 µM Fe-Na-EDTA. The seedlings were grown for 10 days till the first trifoliate leaves fully expanded and then treated with two P supplies (5 µM and 500 µM P). At 18 d after treatment, roots, stems, leaves and flowers were separately harvested. At 29 d after treatment, young pods and seeds were separately harvested. All tissue samples were stored at −80°C for RNA extraction.

To elucidate the responses of the *GmPTs* to the other nutrient deficiencies, ten-day old seedlings precultured in full-strength nutrient solution as described above were treated under −N, −P −K, and -Fe conditions for 14 days, respectively. For −N treatment, KNO_3_, Ca(NO_3_)_2_ and (NH_4_)_6_Mo_7_O_24_ was replaced by 1500 µM K_2_SO_4_, 2000 µM CaSO_4_ and 0.25 µM Na_2_MoO_4_, respectively. For -K treatment, KNO_3_ and KH_2_PO_4_ was replaced by 1500 µM Ca(NO_3_)_2_ and 500 µM NaH_2_PO_4_, respectively. For -Fe treatment, Fe-EDTA was fully withdrawn. Plants continuously grown in the full-strength nutrient solution were sampled as control (CK). Each treatment had three biological replicates. Leaves and roots were separately collected for total RNA extraction and qRT-PCR analysis.

### Quantitative Real-time RT-PCR Analysis

For qRT-PCR analysis, the soybean housekeeping gene *TefS1* (encodes the elongation factor EF-1a: X56856) was used as a reference gene. The optimal primer sequences for *GmPTs* and *TefS1* were listed in Table S2. Total RNA was extracted from soybean plants using RNAiso™ Plus reagent (TaKaRa) according to the manufacturer’s instructions. RNA samples were treated with RNase-free DNaseI (Invitrogen) to remove the contaminating genomic DNA before synthesizing the first strand cDNA using the MMLV-reverse transcriptase (Promega, USA) according to the protocol from the supplier. qRT-PCR was carried out in a 20 µL volume containing 2 µL 1∶50 diluted reverse transcription product, 0.2 µM primers, and 10 µL SYBR® Premix EX Taq™ (TaKaRa). All the reactions were done on a DNA Engine Opticon 2 Continuous Fluorescence Detection System (MJ Research Inc., Waltham, MA). Reaction conditions for thermal cycling were: 95°C for 1 min, 40 cycles of 95°C for 15 s, 58–60°C for 15 s, and 72°C for 30 s. The annealing temperature (58–60°C) was adjusted to suit the amplification of individual *GmPTs*. Fluorescence data were collected during the cycle at 72°C.

### Data Analysis

All the data were analyzed statistically using Microsoft Excel 2003 (Microsoft Company, USA) for calculating mean and standard error. Comparisons of gene expressions in different tissues or responses to different nutrient deficiencies were performed using the student *t*-test in the Microsoft Excel 2003.

## Supporting Information

Figure S1 **Alignment of amino acid sequences of the Pht1 family phosphate transporters in soybean.** Sequence alignment was performed with the ClustalW program [65]. Identical and similar amino acids are shaded in black and grey, respectively. The membrane spanning domains of GmPTs predicted by TopPred (http://www.cbib.u-bordeaux2.fr/pise/toppred.html) are under lined and numbered by roman numerals (I–XII).
(TIF)

Figure S2 **Schematic diagram of intron/exon structure of** ***GmPT*** **genes.** The thin line represents the introns and the open boxes indicate the exons of the respective genes.
(TIF)

Figure S3 **Kinetic growth profiles of yeast transformants.** Yeast strains, including WT, empty vector control or Yp112-GmPTs transformants, grew in the logarithmic phase (OD600≈0.90), then 100 µL different yeast cells were subjected to 3.5 mL YNB liquid medium with different Pi concentrations and incubated at 30°C, OD 600 were measured every 5 hours up to 25 hours. The Pi concentrations were selected according to their *Km* values.
(TIF)

Table S1 **Percentage of protein sequences identity among the 14 soybean phosphate transporters in Pht1 family.**
(DOC)

Table S2 **Genes and gene-specific primers used for quantitative real-time PCR experiments.**
(DOC)

Table S3 **Expression pattern of soybean GmPTs in the common used tissues in reference 37, 38. The numbers presented in the table are normalized Illumina-Solexa reads number coming from the according experiment.**
(DOC)

Table S4 **Primers used to generate the expression vectors in yeast complementary assays (restriction site sequences are underlined).**
(DOC)

Table S5 **Primers used to generate DNA sequences of GmPTs.**
(DOC)

Text S1 **Protein sequences of GmPTs.**
(TXT)

Text S2 **Nucleic acid sequences of** ***GmPTs.***
(TXT)
