# Supplementary figures and images for: Functional Characterization of 14 Pht1 Family Genes in Yeast and Their Expressions in Response to Nutrient Starvation in Soybean
Source: PLoS One. 2012 Oct 25;7(10):e47726. doi: 10.1371/journal.pone.0047726 (PMC3485015; doi:10.1371/journal.pone.0047726)

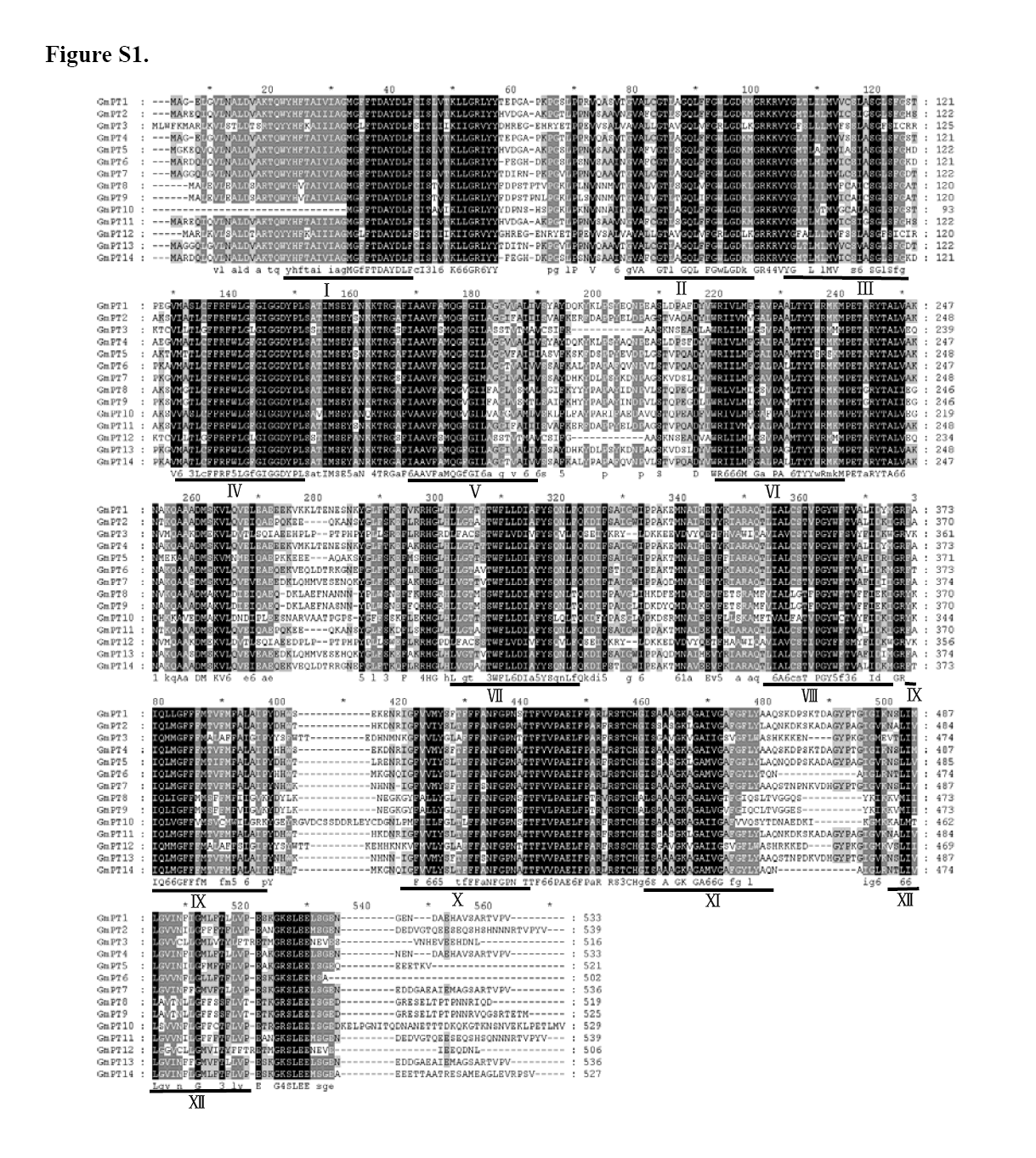

Supplement: Figure S1 — Alignment of amino acid sequences of the Pht1 family phosphate transporters in soybean. Sequence alignment was performed with the ClustalW program [65]. Identical and similar amino acids are shaded in black and grey, respectively. The membrane spanning domains of GmPTs predicted by TopPred (http://www.cbib.u-bordeaux2.fr/pise/toppred.html) are under lined and numbered by roman numerals (I–XII). (TIF) [file pone.0047726.s001.tif]

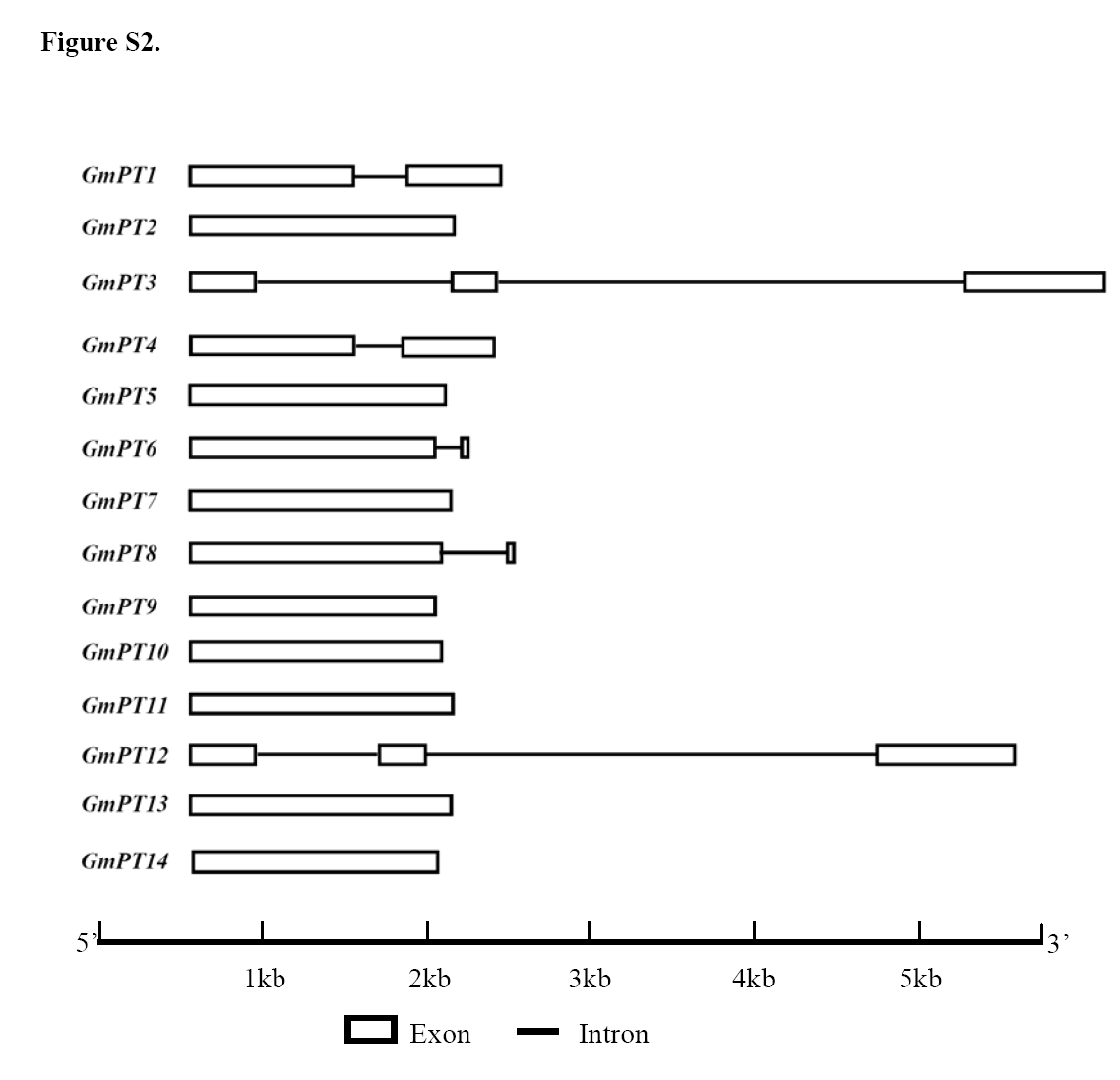

Supplement: Figure S2 — Schematic diagram of intron/exon structure of GmPT genes. The thin line represents the introns and the open boxes indicate the exons of the respective genes. (TIF) [file pone.0047726.s002.tif]

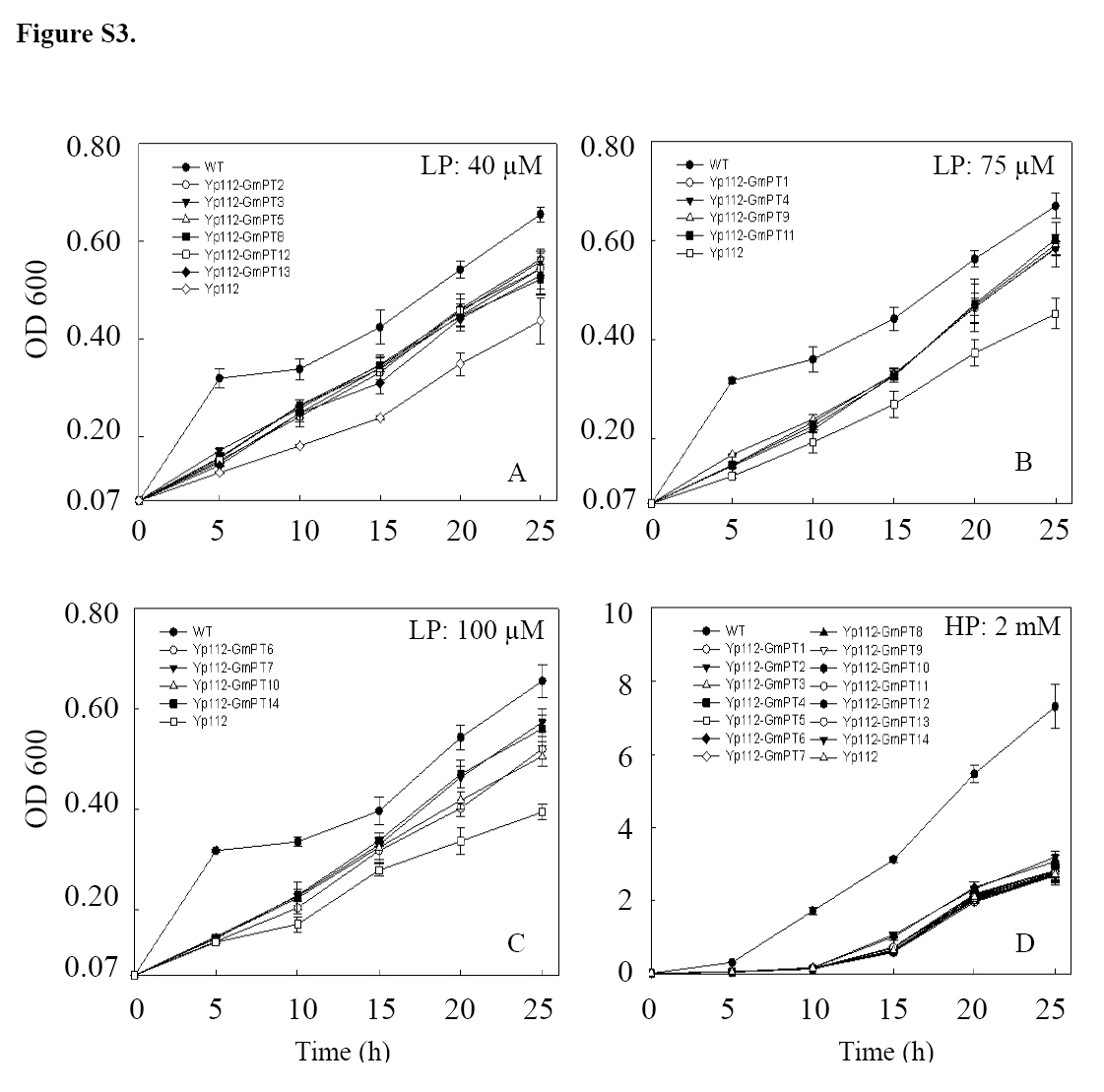

Supplement: Figure S3 — Kinetic growth profiles of yeast transformants. Yeast strains, including WT, empty vector control or Yp112-GmPTs transformants, grew in the logarithmic phase (OD600≈0.90), then 100 µL different yeast cells were subjected to 3.5 mL YNB liquid medium with different Pi concentrations and incubated at 30°C, OD 600 were measured every 5 hours up to 25 hours. The Pi concentrations were selected according to their Km values. (TIF) [file pone.0047726.s003.tif]
