# Supplementary material for: Functional Characterization of 14 Pht1 Family Genes in Yeast and Their Expressions in Response to Nutrient Starvation in Soybean
Source: PLoS One. 2012 Oct 25;7(10):e47726. doi: 10.1371/journal.pone.0047726 (PMC3485015; doi:10.1371/journal.pone.0047726)
Supplement: Table S1 — Percentage of protein sequences identity among the 14 soybean phosphate transporters in Pht1 family. (DOC) [file pone.0047726.s004.doc]

**Table S1. Percentage of protein sequences identity among the 14 soybean phosphate transporters in Pht1 family.**

| % ident. | GmPT1 | GmPT2 | GmPT3 | GmPT4 | GmPT5 | GmPT6 | GmPT7 | GmPT8 | GmPT9 | GmPT10 | GmPT11 | GmPT12 | GmPT13 |
| --- | --- | --- | --- | --- | --- | --- | --- | --- | --- | --- | --- | --- | --- |
| GmPT2 | 78.17 |  |  |  |  |  |  |  |  |  |  |  |  |
| GmPT3 | 50.80 | 54.51 |  |  |  |  |  |  |  |  |  |  |  |
| GmPT4 | 97.04 | 79.10 | 53.80 |  |  |  |  |  |  |  |  |  |  |
| GmPT5 | 77.74 | 87.72 | 52.60 | 77.93 |  |  |  |  |  |  |  |  |  |
| GmPT6 | 81.03 | 83.17 | 55.31 | 80.83 | 79.76 |  |  |  |  |  |  |  |  |
| GmPT7 | 85.71 | 78.11 | 54.00 | 85.71 | 76.78 | 79.84 |  |  |  |  |  |  |  |
| GmPT8 | 58.91 | 64.05 | 51.46 | 58.72 | 60.83 | 62.48 | 59.69 |  |  |  |  |  |  |
| GmPT9 | 59.30 | 64.27 | 51.25 | 58.72 | 61.22 | 62.87 | 59.69 | 94.66 |  |  |  |  |  |
| GmPT10 | 61.25 | 61.43 | 47.63 | 61.04 | 58.58 | 62.98 | 59.08 | 64.36 | 63.73 |  |  |  |  |
| GmPT11  GmPT12  GmPT13  GmPT14 | 78.17  52.91  86.46  81.18 | 99.27  54.12  78.11  82.45 | 54.11  93.89  53.60  55.31 | 79.10  53.11  86.46  80.98 | 87.91  51.49  76.78  79.53 | 83.17  54.51  80.43  98.23 | 77.92  54.64  98.16  80.00 | 63.83  50.31  59.30  62.18 | 64.05  50.10  59.30  62.57 | 61.64  49.89  59.08  61.39 | 53.72  77.92  82.45 | 54.64  54.34 | 80.59 |
