# Supplementary material for: Functional Characterization of 14 Pht1 Family Genes in Yeast and Their Expressions in Response to Nutrient Starvation in Soybean
Source: PLoS One. 2012 Oct 25;7(10):e47726. doi: 10.1371/journal.pone.0047726 (PMC3485015; doi:10.1371/journal.pone.0047726)
Supplement: Table S2 — Genes and gene-specific primers used for quantitative real-time PCR experiments. (DOC) [file pone.0047726.s005.doc]

**Table S2.** **Genes and gene-specific primers used for quantitative real-time PCR experiments.**

| *GmPTs* | Sequence (5’ to 3’) | Amplicon size (bp) |
| --- | --- | --- |
| *GmPT1* | F: CAGGTTCTGGCTAGGGTTTG  R: ACATAGTCAAATGCGGGGTC | 243 |
| *GmPT2* | F: GACATAGCGCGAAATCTGTC  R: CAAACACGGCCGCAATGAAG | 150 |
| *GmPT3* | F: ACAAGAAGACAAGAGGGTCG  R: AACCGAGCATGAGAATCAAC | 162 |
| *GmPT4* | F: AGGTGCACCAAAGCCGGGAACT  R: TGGCCATGACACCCTCTGCA | 215 |
| *GmPT5* | F: GAACACTTTCAGGGCAACTC  R: GTCATCACAGTCTTTGCATCG | 145 |
| *GmPT6* | F: CTGCTCACATACTATTGGCGT  R: GTCCAACAGGAACCAAGTAAC | 249 |
| *GmPT7* | F: TGACCACAAGTACGATCTTCC  R: CGCCAATAGTAGGTAAGAGCA | 129 |
| *GmPT8* | F: TCATTTTCGCGGGTTTAGTC  R: GCTTGCTTCACGTTTCCTTC | 232 |
| *GmPT9* | F: ATGTTTAACTGTGGGCGGCG  R: CCCTATTATTGGGCGTCGGT | 182 |
| *GmPT10* | F: GGACTCCCGAATGAATGCTA  R: AGCTGCAGTCAACTCCCCTA | 218 |
| *GmPT11* | F: GAGCACTCCCAGCTGCATTG  R: GGCGACTGAGGAAGTCCTTG | 204 |
| *GmPT12* | F: GGACCACCAAGGAACATCATAA  R: CCCAGAAATGCCATGACAAC | 155 |
| *GmPT13* | F: GAGGGGCATTCATTGCTGCA  R: AGCGAATCCACCTTCGAACCT | 151 |
| *GmPT14* | F: GAGCAATTGGACACAAGAAG  R: TCCAACAGGAACCAAGTAGT | 110 |
| *TefS1* | F: TGCAAAGGAGGCTGCTAACT | 200 |
|  | R: CAGCATCACCGTTCTTCAAA |  |
