# Supplementary material for: Functional Characterization of 14 Pht1 Family Genes in Yeast and Their Expressions in Response to Nutrient Starvation in Soybean
Source: PLoS One. 2012 Oct 25;7(10):e47726. doi: 10.1371/journal.pone.0047726 (PMC3485015; doi:10.1371/journal.pone.0047726)
Supplement: Table S3 — Expression pattern of soybean GmPTs in the common used tissues in reference 37, 38. The numbers presented in the table are normalized Illumina-Solexa reads number coming from the according experiment. (DOC) [file pone.0047726.s006.doc]

**Table S3. Expression pattern of soybean GmPTs in the common used tissues in Reference 37, 38. The numbers presented in the table are normalized Illumina-Solexa reads number coming from the according experiment.**

| Gene name | Reference 37  Libault et al., Plant J, 2010 (63): 86-99 | | | |  | Reference 38  Severin et al., BMC Plant Biol, 2010 (10): 160 | | | | | | | | |
| --- | --- | --- | --- | --- | --- | --- | --- | --- | --- | --- | --- | --- | --- | --- |
|  | Leaf | Flower | Green Pod | Root |  | Young leaf | Flower | One cm Pod | | Seed  10 DAF | | Root | |  |
| *GmPT1* | 0 | 0 | 0 | 2324 |  | 0 | 0 | 0 | | 0 | | 139 | |  |
| *GmPT2* | 9 | 93 | 11 | 266 |  | 0 | 3 | 0 | | 0 | | 29 | |  |
| *GmPT3* | 21 | 2 | 2 | 48 |  | 0 | 0 | 0 | | 0 | | 12 | |  |
| *GmPT4* | 2 | 0 | 0 | 2023 |  | 0 | 0 | 0 | | 0 | | 212 | |  |
| *GmPT5* | 0 | 212 | 4 | 19 |  | 0 | 18 | 0 | | 0 | | 3 | |  |
| *GmPT6* | 21 | 10 | 22 | 1 |  | 2 | 1 | 2 | | 1 | | 0 | |  |
| *GmPT7* | 416 | 363 | 112 | 562 |  | 4 | 31 | 8 | | 2 | | 52 | |  |
| *GmPT8* | 0 | 0 | 0 | 0 |  | 0 | 0 | 0 | | 0 | | 0 | |  |
| *GmPT9* | 0 | 0 | 0 | 0 |  | 0 | 0 | 0 | | 0 | | 0 | |  |
| *GmPT10* | 0 | 0 | 0 | 0 |  | 0 | 0 | 0 | | 0 | | 0 | |  |
| *GmPT11* | 17 | 25 | 0 | 7 |  | 0 | 1 | 0 | | 0 | | 3 | |  |
| *GmPT12* | 7 | 18 | 9 | 52 |  | 0 | 1 | 1 | | 0 | | 3 | |  |
| *GmPT13* | 128 | 166 | 9 | 98 |  | 3 | 10 | 4 | | 1 | | 8 | |  |
| *GmPT14* | 3 | 0 | 0 | 0 |  | 0 | 0 | | 0 | | 0 | | 0 | |
