# Supplementary material for: Functional Characterization of 14 Pht1 Family Genes in Yeast and Their Expressions in Response to Nutrient Starvation in Soybean
Source: PLoS One. 2012 Oct 25;7(10):e47726. doi: 10.1371/journal.pone.0047726 (PMC3485015; doi:10.1371/journal.pone.0047726)
Supplement: Table S4 — Primers used to generate the expression vectors in yeast complementary assays (restriction site sequences are underlined). (DOC) [file pone.0047726.s007.doc]

**Table S4. Primers used to generate the expression vectors in yeast complementary assays (restriction site sequences underlined).**

| *GmPTs* | Sequence (5’ to 3’) | Incorporated restriction sites |
| --- | --- | --- |
| *GmPT1* | F: atcgGCGGCCGCATGGCTGGAGAATTGGGAGTT  R: atcgAGATCTTCAAACAGGAACTGTCCTAGCA | *Not*Ⅰ  *Bgl*Ⅱ |
| *GmPT2* | F: atcgGCGGCCGCATGGCCAGGGAGCAAATTCAG  R: atcgGGATCCATCCTAAACATATGGAACAGTTC | *Not*Ⅰ  *Bam*HⅠ |
| *GmPT3* | F: atcgGCGGCCGCATGTTGTGGTTCAAAATGGCGAG  R: atcgAGATCTTCAAAGATTATCATGCTCTTCAAC | *Not*Ⅰ  *Bgl*Ⅱ |
| *GmPT4* | F: atcgGCGGCCGCATGGCTGGAGAACTTGGAGTG  R: atcgAGATCTTCAAACAGGAACTGTCCTAGCA | *Not*Ⅰ  *Bgl*Ⅱ |
| *GmPT5* | F: atatGCGGCCGCATGGGGAAGGAGCAAGTTCAGG  R: gcgcGGATCCTTACACCTTGGTCTCCTCTTCTTG | *Not*Ⅰ  *Bam*HⅠ |
| *GmPT6* | F: atcgGCGGCCGCATGGCCAGGGATCAGTTGC  R: atcgGGATCCGCTAAGCAGACATCTCCTCCAGG | *Not*Ⅰ  *Bam*HⅠ |
| *GmPT7* | F: atcg GCGGCCGCATGGCGGGAGGACAACTAGGA  R: atcg GGATCCTTAAACTGGAACCGTCCTAGCAG | *Not*Ⅰ  *Bam*HⅠ |
| *GmPT8* | F: atcg GCGGCCGCATGGCACTGGAAGTGCTTGAAG  R: atcgGGATCCCTAGTCCTGAATCCTATTATTGGG | *Not*Ⅰ  *Bam*HⅠ |
| *GmPT9* | F: atcg GCGGCCGCATGGCATTGGAAGTGCTTGAAG  R: atcg GGATCCTCACATCGTCTCAGTCCTTGAT | *Not*Ⅰ  *Bam*HⅠ |
| *GmPT10* | F: atcgGCGGCCGCATGGGGTTCTTCACCGATGC  R: atcgGGATCCCTAAACCATCAAGGTTTCTGGAAG | *Not*Ⅰ  *Bam*HⅠ |
| *GmPT11* | F: atcgGCGGCCGCATGGCCAGGGAGCAAATTCA  R: atcgGGATCCCTAAACATATGGAACTGTTCTATTG | *Not*Ⅰ  *Bam*HⅠ |
| *GmPT12* | F: atcgGCGGCCGCATGGCTAGGTTGAAGGTGTTGTC  R: atcgGCGGCCGCTCAAAGATTATCTTGCTCTTCAATC | *Not*Ⅰ  *Not*Ⅰ |
| *GmPT13* | F: atcgGCGGCCGCATGGCAGGAGGACAACTAGGAG  R: atcgGCGGCCGCTTAAACTGGAACCGTCCTAGCAG | *Not*Ⅰ  *Not*Ⅰ |
| *GmPT14* | F: atcgGCGGCCGCATGGCTAGGGATCAGTTGCAAG  R: atcgGGATCCTTATACAGAAGGCCTAACTTCTAGACC | *Not*Ⅰ  *Bam*HⅠ |
