# Supplementary material for: Functional Characterization of 14 Pht1 Family Genes in Yeast and Their Expressions in Response to Nutrient Starvation in Soybean
Source: PLoS One. 2012 Oct 25;7(10):e47726. doi: 10.1371/journal.pone.0047726 (PMC3485015; doi:10.1371/journal.pone.0047726)
Supplement: Table S5 — Primers used to generate DNA sequences of GmPTs. (DOC) [file pone.0047726.s008.doc]

**Table S5. Primers used to generate DNA sequences of *GmPTs*.**

| *GmPTs* | Sequence (5’ to 3’) |
| --- | --- |
| *GmPT1* | F1: atcgGCGGCCGCATGGCTGGAGAATTGGGAGTT  R1: atcgAGATCTTCAAACAGGAACTGTCCTAGCA |
| *GmPT2* | F: atcgGCGGCCGCATGGCCAGGGAGCAAATTCAG  R: atcgGGATCCATCCTAAACATATGGAACAGTTC |
| *GmPT3* | F1: atcgGCGGCCGCATGTTGTGGTTCAAAATGGCGAG  R1: atcgAGATCTTCAAAGATTATCATGCTCTTCAAC  F2: ATGGCGAGGTTGAAGGTGTTGT  R2: GTCAAGATGTCAACGGAGGCAG  F3: ATATTGGCCAGCTCCACCGTG  R3: GGCGAACGCAACCTGGCATT  F4: AATGCCAGGTTGCGTTCG  R4: TCAAAGATTATCATGCTCTTC |
| *GmPT4* | F: atcgGCGGCCGCATGGCTGGAGAACTTGGAGTG  R: atcgAGATCTTCAAACAGGAACTGTCCTAGCA |
| *GmPT5* | F: atatGCGGCCGCATGGGGAAGGAGCAAGTTCAGG  R: gcgcGGATCCTTACACCTTGGTCTCCTCTTCTTG |
| *GmPT6* | F: atcgGCGGCCGCATGGCCAGGGATCAGTTGC  R1: atcgGGATCCGCTAAGCAGACATCTCCTCCAGG  R2: GGAGCTGTAACAGAGAGCACATGT |
| *GmPT7* | F: atcg GCGGCCGCATGGCGGGAGGACAACTAGGA  R: atcg GGATCCTTAAACTGGAACCGTCCTAGCAG |
| *GmPT8* | F: atcg GCGGCCGCATGGCACTGGAAGTGCTTGAAG  R1: atcgGGATCCCTAGTCCTGAATCCTATTATTGGG  R2: GGTCAGGGGAAGTGAGATACGA |
| *GmPT9* | F: atcg GCGGCCGCATGGCATTGGAAGTGCTTGAAG  R: atcg GGATCCTCACATCGTCTCAGTCCTTGAT |
| *GmPT10* | F: atcgGCGGCCGCATGGGGTTCTTCACCGATGC  R: atcgGGATCCCTAAACCATCAAGGTTTCTGGAAG |
| *GmPT11* | F: atcgGCGGCCGCATGGCCAGGGAGCAAATTCA  R: atcgGGATCCCTAAACATATGGAACTGTTCTATTG |
| *GmPT12* | F1: atcgGCGGCCGCATGGCTAGGTTGAAGGTGTTGTC  R1: atcgGCGGCCGCTCAAAGATTATCTTGCTCTTCAATC  F2: ATGGCTAGGTTGAAGGTGTTG  R2: GGTAGTCTCCTCCAATACCCAGC  F3: ACCGTGACCATGGCAGTGTG  R3: TACGAAGCCATAGGTTCCTTC  F4: GAAGGAACCTATGGCTTCGTA  R4: TAGGTCAGGACCGTGCCTACG |
| *GmPT13* | F: atcgGCGGCCGCATGGCAGGAGGACAACTAGGAG  R: atcgGCGGCCGCTTAAACTGGAACCGTCCTAGCAG |
| *GmPT14* | F: atcgGCGGCCGCATGGCTAGGGATCAGTTGCAAG  R:atcgGGATCCTTATACAGAAGGCCTAACTTCTAGACC |

Note: Primers with underlined restriction site are also used in the yeast complementary assays.
